# Supplementary material for: Disassembly of HIV envelope glycoprotein trimer immunogens is driven by antibodies elicited via immunization
Source: bioRxiv. 2021 Feb 17:2021.02.16.431310. Preprint. [Version 1] doi: 10.1101/2021.02.16.431310 (PMC7899455; doi:10.1101/2021.02.16.431310)
Supplement: Supplement 1 [file media-1.pdf]

Supplementary Materials for

Disassembly of HIV envelope glycoprotein trimer immunogens is driven by antibodies elicited via immunization

Hannah L. Turner, Raiees Andrabi, Christopher A. Cottrell, Sara T. Richey, Ge Song, Sean Callaghan, Fabio Anzanello, Tyson J. Moyer, Wuhbet Abraham, Mariane Melo, Murillo Silva, Nicole Scaringi, Eva G. Rakasz, Quentin Sattentau, Darrell J. Irvine, Dennis R. Burton, Andrew B. Ward*

*Corresponding author. Email: andrew@scripps.edu

Published:

The PDF file includes:

Figs. S1 to S13.

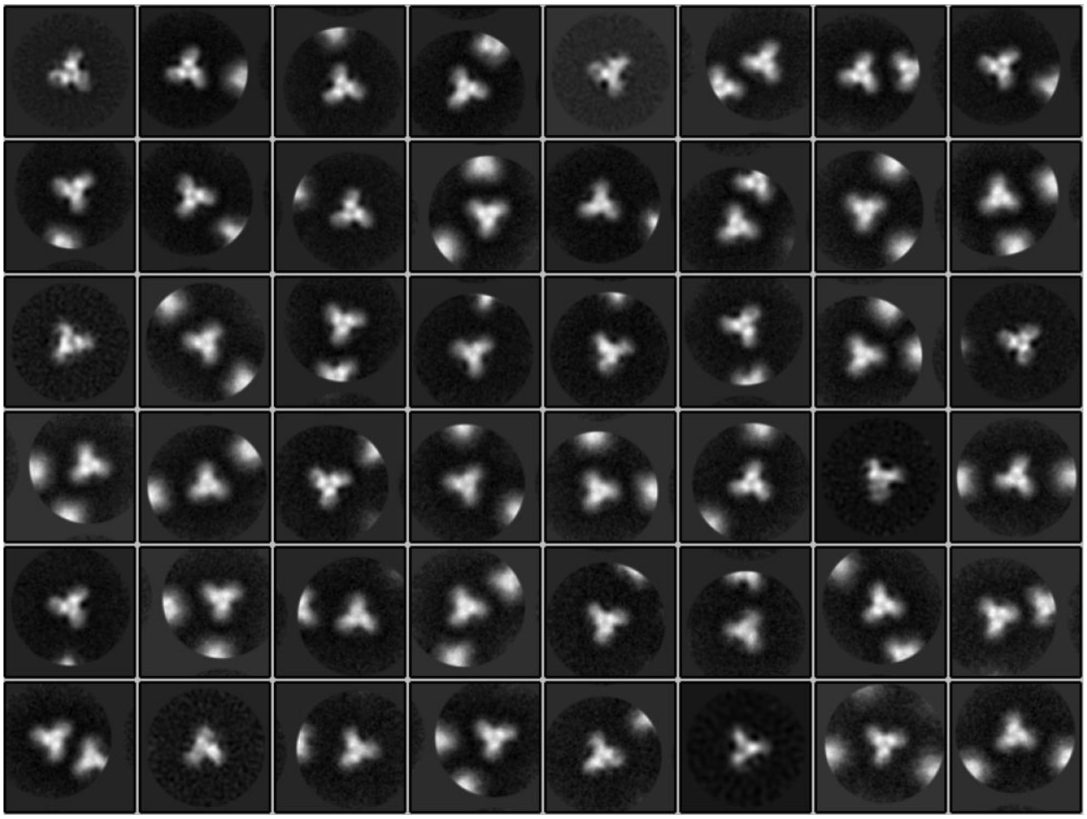

Fig. S1. 2D classes of unliganded MT145KdV5 trimer. Only top views due to orientation bias without ligand.

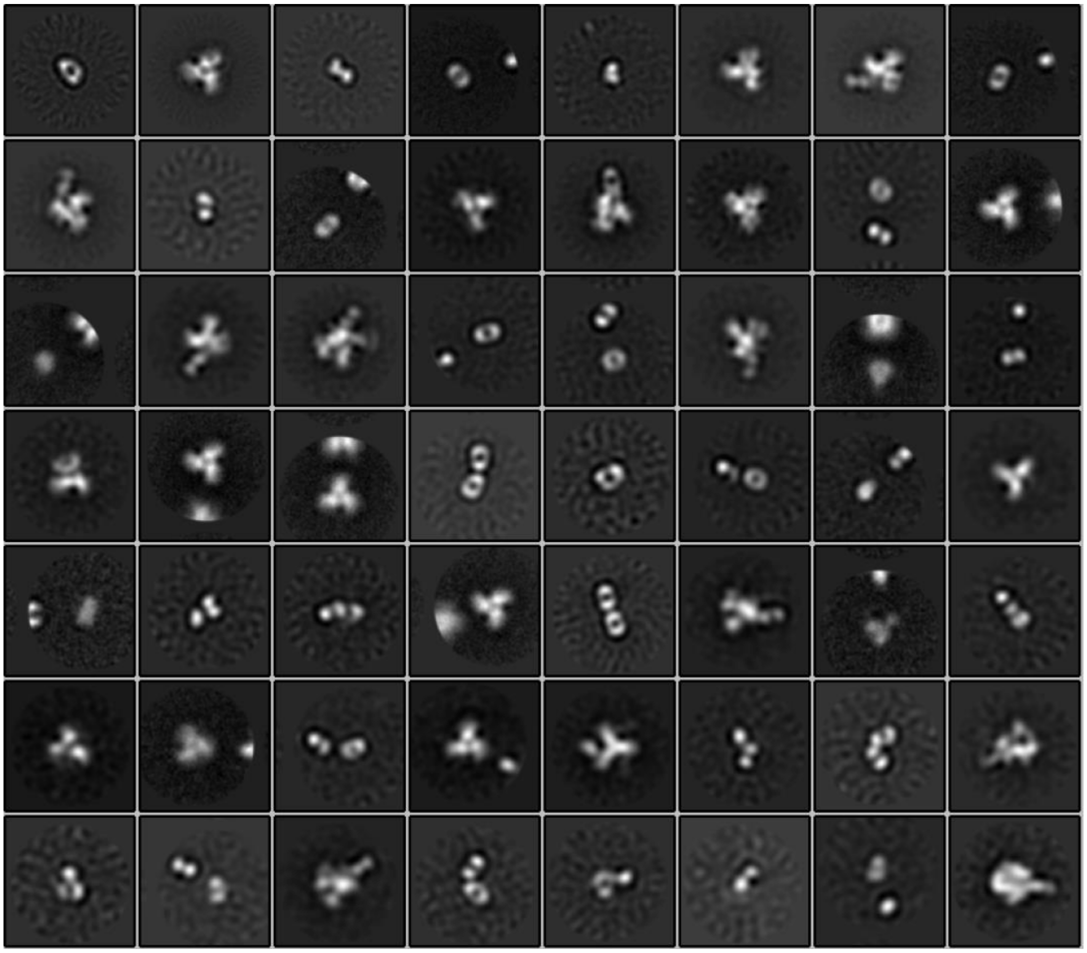

Fig. S2. 2D classes of rhesus macaque 2688 serum in complex with MT145KdV5, incubated for 30 minutes. Initial 2D classes of rhesus macaque 2688 fab-isolated serum 10 weeks after immunization with MT145KdV5 SOSIP pSer. Fab in complex with MT145KdV5 SOSIP.664 and incubated for 30 minutes before being added to nsEM grid.

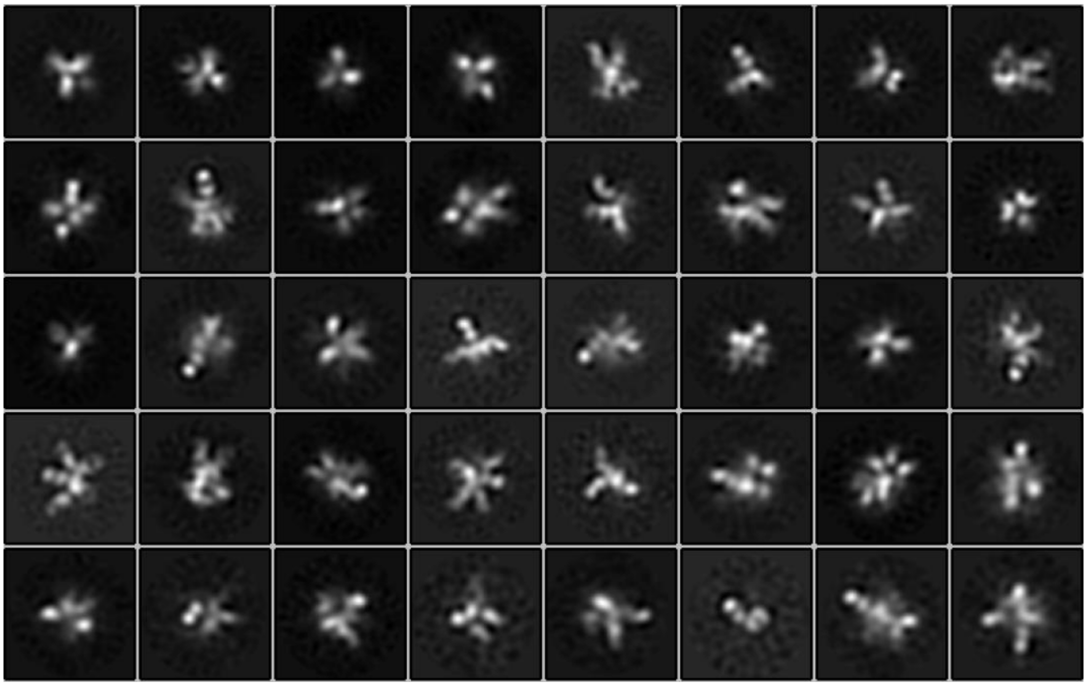

Fig. S3. 2D classes of rhesus macaque 2688 serum in complex with MT145KdV5. Initial 2D classes of rhesus macaque 2688 fab-isolated serum 10 weeks after immunization with MT145KdV5 SOSIP pSer. Fab serum in complex with MT145KdV5 SOSIP.664 and incubated overnight.

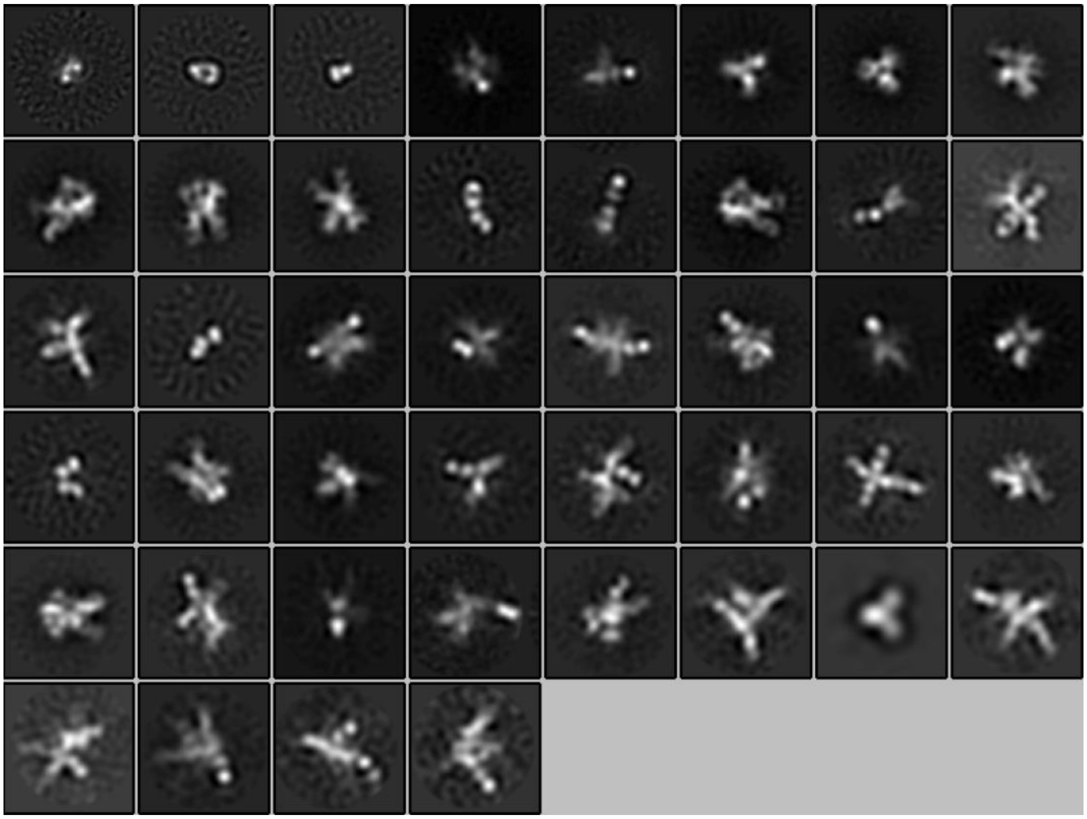

Fig. S4. 2D classes of rhesus macaque BK89 serum in complex with MT145KdV5 SOSIP.664.
Initial 2D classes of rhesus macaque BK89 fab-isolated serum in complex with MT145KdV5 SOSIP.664, 10 weeks after immunization with MT145KdV5 SOSIP.664.

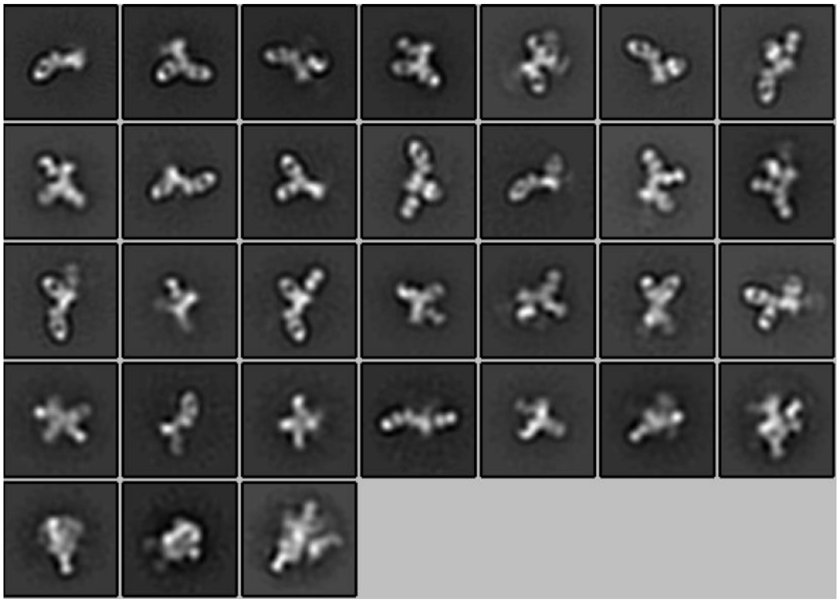

Fig. S5. 2D classes of rabbit K3 serum post 26 weeks immunization. Initial 2D classes of rabbit K3 fab isolated serum after 26 weeks of immunization with BG505-CRF250V1V2-grafter trimer. Fab serum in complex with CRF250 SOSIP.664 Env trimer.

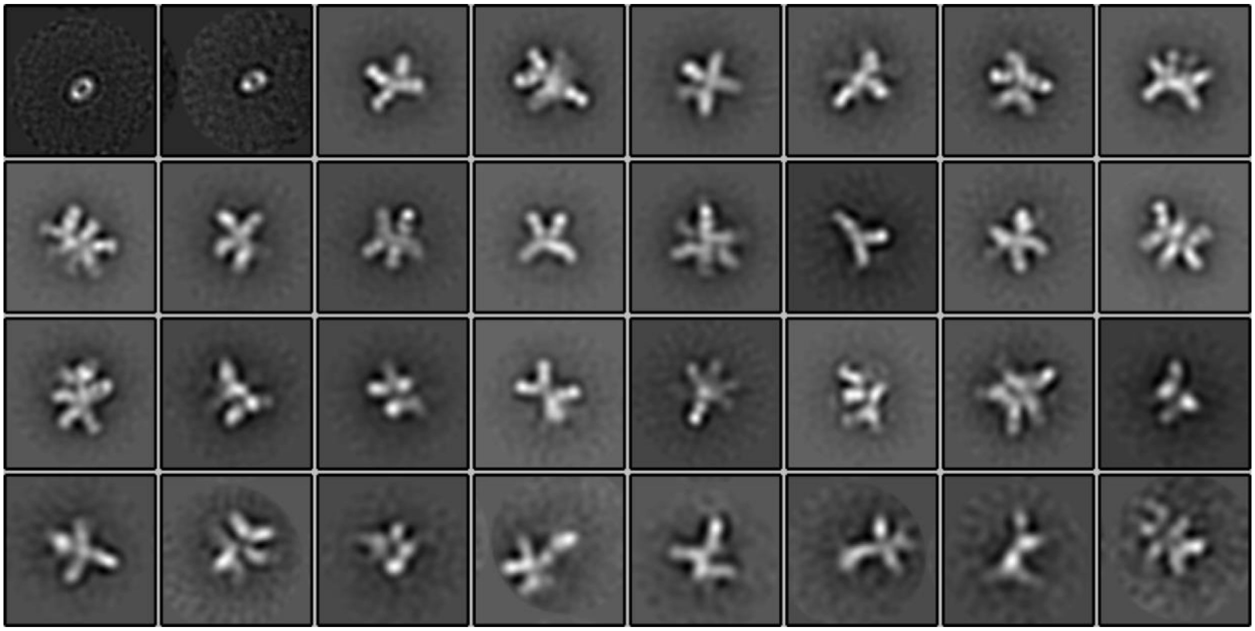

Fig. S6. 2D classes of rabbit 2382 serum immunized with ConM SOSIP.664. Initial 2D classes of rabbit 2382 fab-isolated serum in complex with ConM SOSIP.664, 22 weeks post immunization with ConM SOSIP.664.

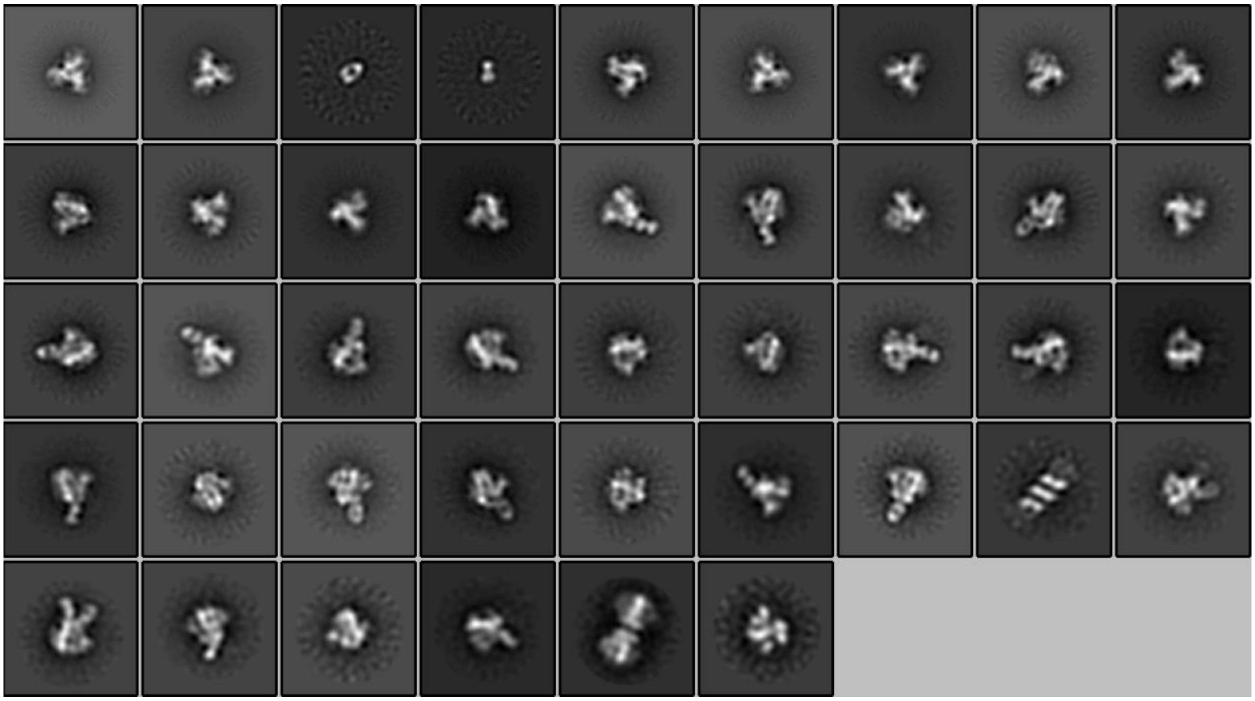

Fig. S7. 2D classes of rabbit 3417 serum two weeks post prime immunization. Initial 2D classes of rabbit 3417 fab-isolated serum two weeks after prime immunization of 20ug BG505 SOSIP.664 liposome i.d. Fab serum in complex with BG505 SOSIP.664.

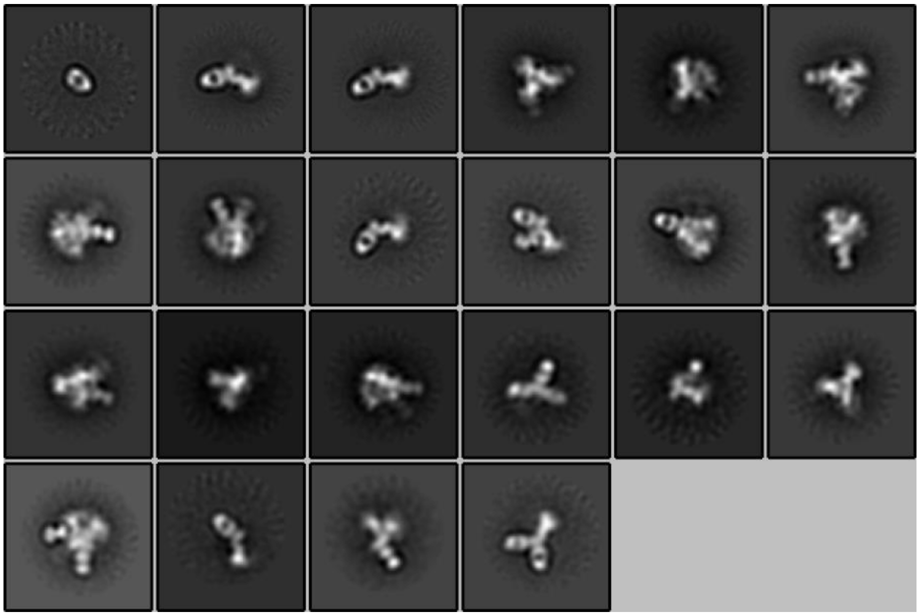

Fig. S8. 2D classes of Rabbit 3417 after boost 1. Initial 2D classes of rabbit 3417 fab isolated serum two weeks after 20ug boost 1 via i.m. and 6 weeks after 20ug prime immunization of BG505 SOSIP.664 liposome i.d. Fab serum in complex with BG505 SOSIP.664

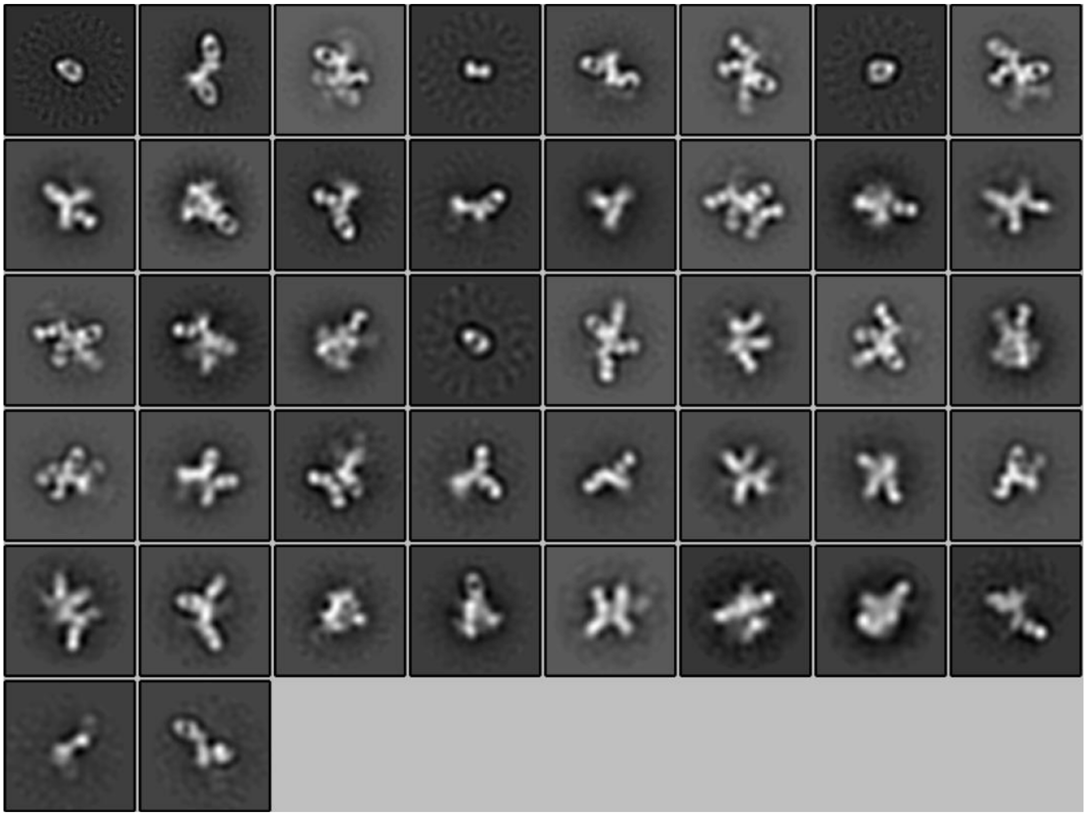

Fig. S9. 2D classes of rabbit M3 serum 26 weeks after immunization with V1V2-grafted CRF250. Initial 2D classes of rabbit M3 fab-isolated serum in complex with CRF250, 26 weeks after being immunized with BG505 CRF250V1V2-grafted trimer.

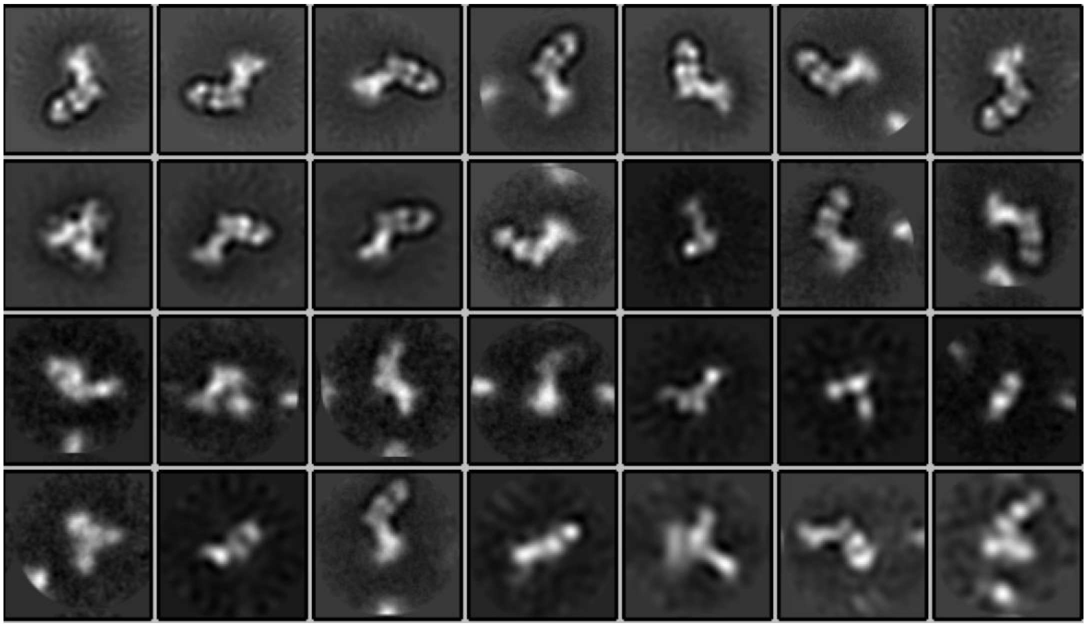

Fig. S10. 2D classes of RM20C in complex with BG505v3. Initial 2D classes of RM20C fab in complex with version 3 of BG505 SOSIP.664. Complex was incubated overnight and placed on a nsEM grid.

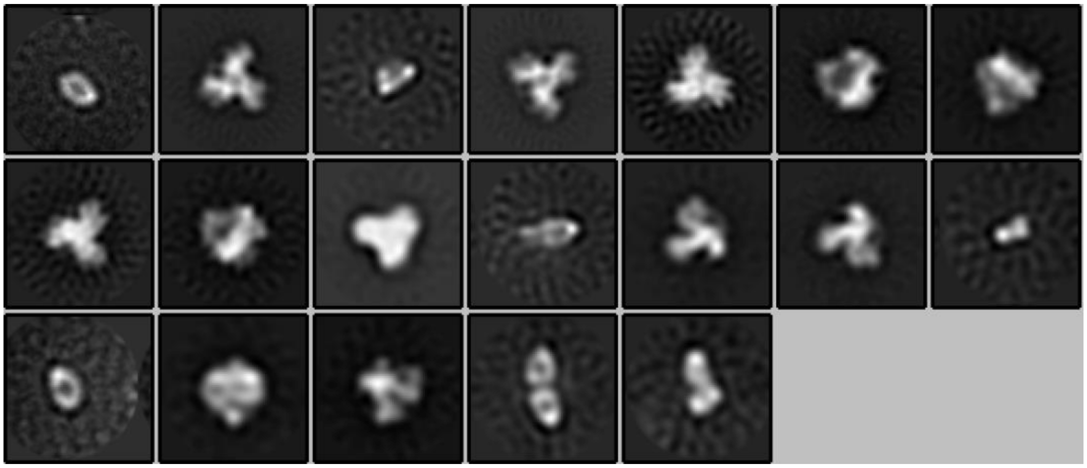

Fig. S11. 2D classes of RM20C in complex with BG505v5.2 CC2. Initial 2D classes of RM20C fab in complex with BG505 SOSIP.664 CC2. Complex was incubated overnight and placed on a nsEM grid.

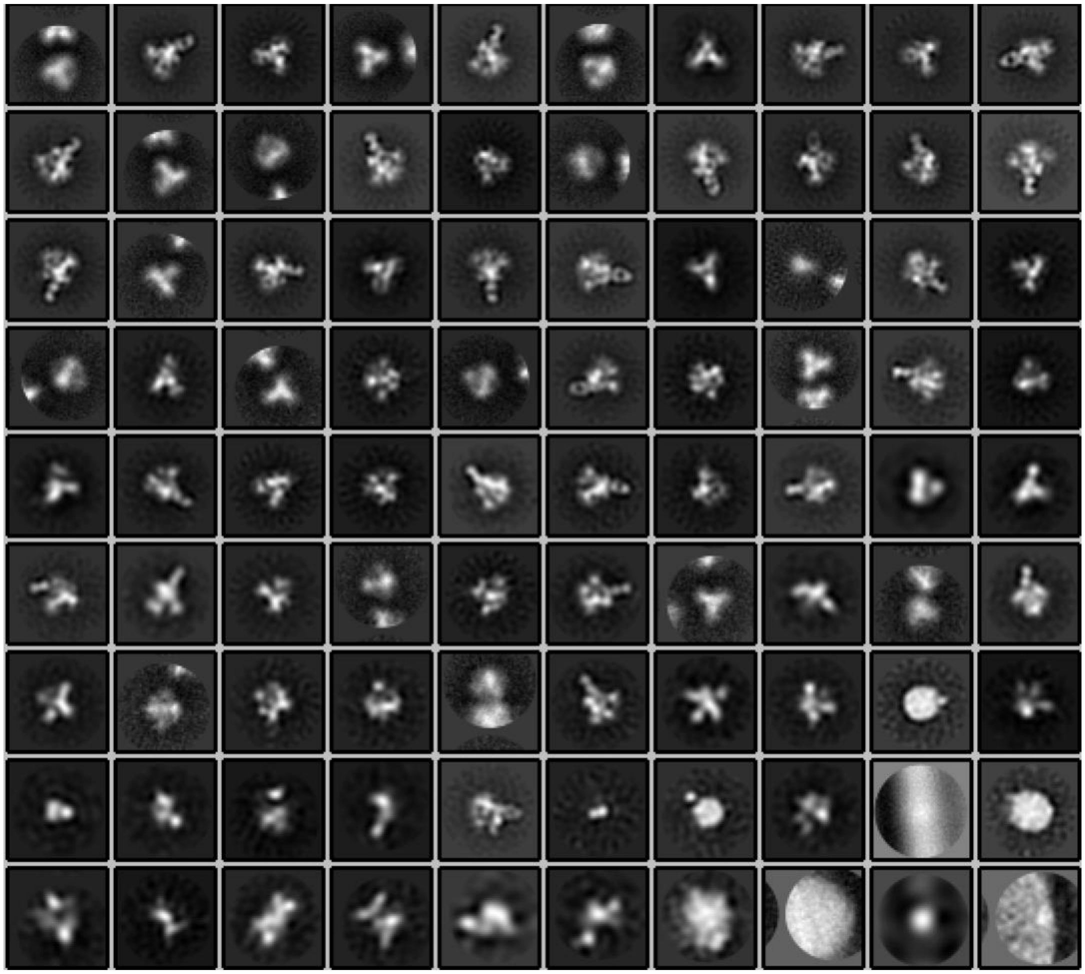

Fig. S12. 2D classes of RM20C in complex with EDC BG505 SOSIP.664. Initial 2D classes of RM20C fab in complex with chemical crosslinked (edc) BG505 SOSIP.664. Complex was incubated overnight and placed on a nsEM grid.

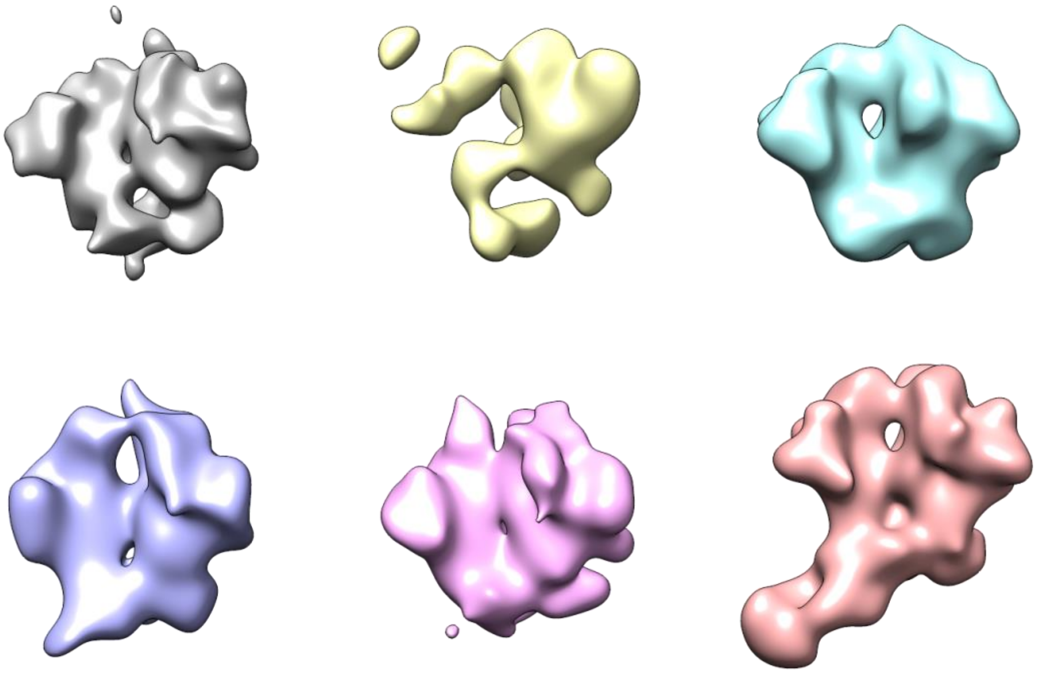

Fig. S13. 3D classes of RM20C in complex with EDC BG505 SOSIP.664. Particles from Class 6 (salmon colored) were selected for 3D refinement.
